# Biocontrol Potential of Endophytic Plant-Growth-Promoting Bacteria against Phytopathogenic Viruses: Molecular Interaction with the Host Plant and Comparison with Chitosan

**DOI:** 10.3390/ijms23136990

**Published:** 2022-06-23

**Authors:** Gul-i-Rayna Shahzad, Alessandro Passera, Giusva Maldera, Paola Casati, Iriti Marcello, Piero Attilio Bianco

**Affiliations:** Department of Agricultural and Environmental Sciences-Production, Landscape, Agroecology, University of Milan, 20133 Milan, Italy; gul.shahzad@unimi.it (G.-i.-R.S.); alessandro.passera@unimi.it (A.P.); giusva.maldera@studenti.unimi.it (G.M.); paola.casati@unimi.it (P.C.); piero.bianco@unimi.it (P.A.B.)

**Keywords:** *Nicotiana benthamiana*, virus quantification, defense-related genes, induced systemic resistance (ISR)

## Abstract

Endophytic plant-growth-promoting bacteria (ePGPB) are interesting tools for pest management strategies. However, the molecular interactions underlying specific biocontrol effects, particularly against phytopathogenic viruses, remain unexplored. Herein, we investigated the antiviral effects and triggers of induced systemic resistance mediated by four ePGPB (*Paraburkholderia fungorum* strain R8, *Paenibacillus pasadenensis* strain R16, *Pantoea agglomerans* strain 255-7, and *Pseudomonas syringae* strain 260-02) against four viruses (Cymbidium Ring Spot Virus—CymRSV; Cucumber Mosaic Virus—CMV; Potato Virus X—PVX; and Potato Virus Y—PVY) on *Nicotiana benthamiana* plants under controlled conditions and compared them with a chitosan-based resistance inducer product. Our studies indicated that ePGPB- and chitosan-treated plants presented well-defined biocontrol efficacy against CymRSV and CMV, unlike PVX and PVY. They exhibited significant reductions in symptom severity while promoting plant height compared to nontreated, virus-infected controls. However, these phenotypic traits showed no association with relative virus quantification. Moreover, the tested defense-related genes (*Enhanced Disease Susceptibility-1* (*EDS1*), *Non-expressor of Pathogenesis-related genes-1* (*NPR1*), and *Pathogenesis-related protein-2B* (*PR2B*)) implied the involvement of a salicylic-acid-related defense pathway triggered by *EDS1* gene upregulation.

## 1. Introduction

Plant viruses are obligate pathogens responsible for nearly half of all known plant diseases, and they pose a major threat to agricultural production worldwide [1,2,3,4]. According to the Ninth Report issued by the International Committee on Taxonomy of Viruses, there are 950 different plant viruses undermining plant health throughout the world [5], and new viruses are discovered yearly. Plant viral infections lead to substantial losses in yield and fruit quality, adversely affecting human wellbeing due to agricultural and economic losses, which may have implications for biodiversity conservation [6]. Most of these viruses have been described as RNA viruses [7], particularly single-stranded, positive-sense RNA viruses [8], and can cause up to 40% losses in important crops [9].

Plant viruses are notoriously difficult to control after the infection takes place [1]. Therefore, containment strategies against viruses are focused on preventing the infection in the first place [10,11,12]. The main techniques that can be employed to prevent the spreading of viruses are the control of vectors, the reduction of inoculum sources, and the use of resistant plants. The use of pesticides against vectors has been the main crop protection strategy in the last years. Although effective in most cases, this approach can have high cost and environmental impact [13,14], might be thwarted by the development of resistance to pesticides [15], and may be ineffective against some virus–vector combinations, depending on their ecology and method of transmission. The reduction of the sources of inoculum is carried out mostly by employing certified, virus-free plant material in fields [16]. The obtainment of resistant plants can be achieved through different methods, such as conventional breeding [17] or biotechnological approaches, such as transgenesis or genome editing [2], but these approaches can take a long time, may not be accepted in some countries, and may be outpaced by the evolution of the pathogen, which especially in the case of viruses, can mutate rapidly.

Another approach that has been researched to pursue the sustainable management of viral diseases is the use of organic products, such as the inoculation of biocontrol agents or resistance inducers. Among organic resistance inducers, chitosan and its derivatives are surely the most well-known and have been utilized as plant growth promoters and biocontrol molecules because of their biodegradability, biocompatibility, and nontoxic nature [18]. Chitosan is derived from chitin in its deacetylated form present in the exoskeleton of crustaceans and is considered the second-most abundant polysaccharide [19]. It has also been reported several times as being capable of triggering defense responses against plant viruses [20,21,22,23,24].

The use of biocontrol agents provides similar benefits, as these microorganisms not only can protect crops from pathogens, but often promote the growth of the host plant through several mechanisms, such as providing nutrients [25] and secreting allelochemicals and plant hormones or hormone-like substances [26,27,28,29,30]. Considering the possibility to control viruses biologically, a specific mention must be made for endophytic plant-growth-promoting bacteria (ePGPB), which have shown the ability to act against a broad spectrum of viral diseases, including Cucumber Mosaic Virus, Tomato Spotted Wilt Virus, Banana Bunchy Top Virus, Tobacco Mosaic Virus, and so on [31,32,33,34,35,36,37,38,39]. As a recent example, a study conducted by Gupta and colleagues [40] demonstrated that the application of *Pantoea agglomerans* strain UN1 24 h prior to the inoculation of Tobacco Mosaic Virus developed systemic antiviral resistance and growth promotion of *Nicotiana glutinosa* and *N*. *tabacum* cv. *Xanthinc* plants. The results obtained thus far have indicated that these bacteria not only act directly against the virus, but also provide indirect antagonism by stimulating an induced systemic resistance (ISR) response in the host [41]. ISR leads toward the activation of several defense-related genes through metabolic pathways mediated by jasmonic acid, ethylene, and salicylic acid [36,42,43], providing protection against a broad range of pests and pathogens, including viruses.

In this scope, the present work aims at characterizing the priming efficacy and mechanism of action of selected candidate ePGPB against plant viruses and comparing their effects to that of a chitosan-based product in the model host plant *Nicotiana benthamiana*. The selected ePGPB strains are *Paraburkholderia fungorum* strain R8, *Pantoea agglomerans* strain 255-7, *Paenibacillus pasadenensis* strain R16, and *Pseudomonas syringae* strain 260-02, the latter two having already been reported as plant-growth-promoting and biocontrol agents against different pathogens [44,45,46].

The ePGPB strain efficacy is tested on four plant viruses: Cymbidium Ring Spot Virus (CymRSV, family Tombusviridae), Cucumber Mosaic Virus (CMV, family Bromoviridae), Potato Virus X (PVX, family Alphaflexiviridae), and Potato Virus Y (PVY, family Potyviridae). All the above-mentioned phytopathogenic viruses are characterized by having a genome constituted by single-stranded, positive-sense RNAs. All these viruses can cause diseases in major crop plants of different families, including Solanaceae, the third-largest and economically important family [47,48,49,50]. CymRSV is a virus with no known vectors and rarely occurs as a major threat to agriculture, whereas CMV, PVX, and PVY have been known to cause severe damage because of having a broad range of hosts [51,52,53] and vectors [54]. The common symptoms include leaf crinkling, mottling, chlorosis, mosaic patterns, stunted growth, necrosis, and ultimately, death of the plant [55].

For conducting the study, we evaluate the phenotypic effect of the treatments with regard to elucidating, particularly, the biocontrol effect of ePGPB on reducing the symptoms caused by the tested viruses in comparison to a chitosan-based product. Furthermore, the priming action of ePGPB strains are evaluated on healthy plants by the expression levels of defense-related genes to have a clearer understanding of the molecular basis behind the observed phenotype.

## 2. Results

### 2.1. Effect of ePGPB Strains against Target Viruses

The evaluation of an in planta bioassay based on the ability of the ePGPB strains to reduce the incidence of virus infection and to enhance the plant growth (with or without the presence of virus infection) had variable effects, depending on both the ePGPB strains and the viruses. The phenotypic evaluation was performed based on the measurements taken during the experiment, i.e., (i) plant heights, (ii) percentage of symptomatic leaves due to systemic infection, and (iii) symptom severity at different time points, each of which is presented in the following paragraphs.

The molecular quantification of the virus was based on the readings taken from the Log_10_ 2^−ΔΔCT^ values of the virus relative quantification at T3 for CymRSV and at T2 for CMV and PVY. The quantification of PVX was not possible, as all the plants were dead at the time of sample collection. All the above-mentioned comparisons were made between virus-infected plants with and without each ePGPB strain and CHI treatment. The NT-C and CHI-C treatments were only included in the plant height parameter, as they were not infected with the viruses and, therefore, showed no symptoms.

#### 2.1.1. Plant Height: A Growth Promotion Parameter

**Figure 1:**
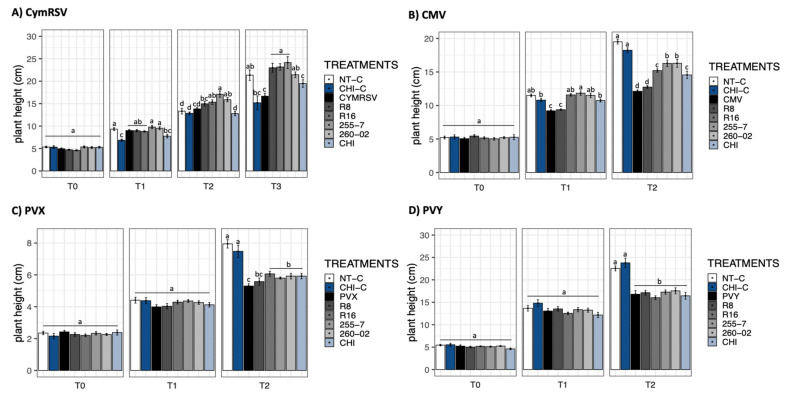
Bar graphs reporting the mean values of *N*. *benthamiana* plant height in cm. (**A**) CymRSV, (**B**) CMV, (**C**) PVX, and (**D**) PVY display the measurements taken at days post-inoculation (dpi) intervals represented as T0 = virus inoculation, T1 = 5 dpi, T2 = 7 dpi, and T3 = 12 dpi. Each bar represents a different treatment: nontreated, healthy control (NT-C), chitosan healthy reference control (CHI-C), nontreated, virus-infected plants (CymRSV, CMV, PVX, and PVY), virus-infected plants treated with either strain R8 (*Paraburkholderia*), R16 (*Paenibacillus*), 255-7 (*Pantoea*), 260-02 (*Pseudomonas*), or a chitosan-based product (CHI). Error bars indicate standard error. Different letters (a–d) indicate statistically significant differences (*p* < 0.05) in the results of a Kruskal–Wallis nonparametric test followed by *p*-adjusted values using Benjamini and Hochberg (BH) methods.

The measurement of plant height showed no significant differences at T0, regardless of the considered combination of treatment and virus (Figure 1).

Regarding plants inoculated with CymRSV at T1, a slight but statistically significant reduction in height was registered for chitosan-treated control plants (CHI-C) and for CymRSV-infected plants treated with CHI (Figure 1A) in comparison with other treatments and, especially, the nontreated control (NT-C). At T2, the differences in heights between treatments became greater, leading to the results visible at T3. In this last observation, all the ePGPB strains gave an increase in plant heights compared to the nontreated, CymRSV-infected plants (CymRSV), restoring heights comparable to those of noninfected plants (NT-C), whereas the chitosan-treated plants produced similar heights in both noninfected and CymRSV-infected plants (Figure 1A).

For CMV, a similar trend was registered: at T1, the difference in heights was less pronounced but already showed a trend that was mostly confirmed at T2 (Figure 1B). Interestingly, the tallest plants were those that were not treated with ePGPB or infected with CMV (NT-C and CHI-C), while the lowest height was registered for the nontreated, CMV-infected plants. Apart from strain R8, all the other treatments, both ePGPB- and chitosan-based, allowed the plants to achieve greater heights, even if not comparable to those of healthy plants (Figure 1B).

The observation made for PVX showed no statistical differences at T1, but a quite visible difference was observed at T2 (Figure 1C). With similar findings to CMV-infected plants at T2, the tallest heights were observed in both noninfected plant groups (NT-C and CHI-C), whereas the lowest heights were observed in the treatments of PVY-infected plants and R8-inoculated, PVY-infected plants. All the other treatments (R16, 255-7, 260-02, and CHI) showed significantly greater heights (Figure 1C).

A similar trend to PVX was observed in the plants inoculated with PVY at T1 (Figure 1D). However, at T2, none of the treatments from both ePGPB and chitosan plants showed any significantly different results in the presence of PVY infection. Nevertheless, significantly different and tallest heights were observed only in noninfected plants with either chitosan-treated (CHI-C) or nontreated (NT-C) plants (Figure 1D).

#### 2.1.2. Systemic Symptomatic Leaves and Symptom Severity: Biocontrol Parameters

**Figure 2:**
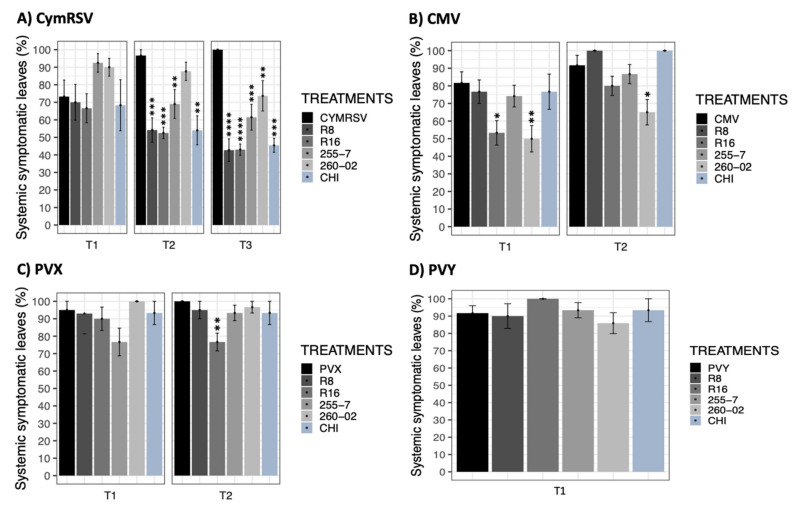
Bar graphs reporting the results for mean percentage values of systemic symptomatic *N*. *benthamiana* leaves. (**A**) CymRSV, (**B**) CMV, (**C**) PVX, and (**D**) PVY display the measurements taken at days post-inoculation (dpi) intervals represented as T1 = 5 dpi, T2 = 7 dpi, and T3 = 12 dpi. Each bar represents a different treatment: nontreated, virus-infected plants (CymRSV, CMV, PVX, and PVY) and virus-infected plants treated with either strain R8 [*Paraburkholderia*], R16 [*Paenibacillus*], 255-7 [*Pantoea*], 260-02 [*Pseudomonas*], or a chitosan-based product (CHI). Error bars indicate standard error. Asterisks indicate significant difference among results according to a nonparametric Wilcoxon rank-sum test followed by Bonferroni methods compared with virus-infected, nontreated plants (* for *p* < 0.05, ** for *p* <0.01, *** for *p* <0.001, and **** for *p* = 0.000).

The percentage of systemic infection of the leaves was evaluated as a parameter that described the ability of the virus to move inside the infected host plant. The results obtained from this parameter were compared through a statistical analysis using a nonparametric Wilcoxon rank-sum test followed by *p*-adjusted values based on Bonferroni methods using the target viruses as the reference of comparison. Based on the overall obtained results, it was observed that all the viruses (CymRSV, CMV, PVX, and PVY) spread to most of the leaves of the plants (systemic infected leaf percentage > 90%) when no treatment was applied (Figure 2).

For CymRSV, there were no significant differences between the percentages of systemic infected leaves in different treatments at T1 despite the great difference in the actual values (ranging from 67 to 92%). At the following timepoints, the number of systemic infected leaves consistently rose in the nontreated, CymRSV-infected plants to 100% at T3 (Figure 2A). In contrast, the percentage decreased notably in all the ePGPB-treated plants, becoming around 40% for treatments with R8, R16, and CHI and 60 to 70% for treatments with the 255-7 and 260-02 strains, indicating the influence of the inoculants to restrict the virus spread to the newer leaves that were produced. The percentage of systemic infected leaves for CMV showed no statistically significant difference between treatments, except for strains R16 and 260-02 at T1. Moreover, the 260-2 strain was consistently able to give lower percentages of infected leaves, even at T2, exhibiting a reduction of around 30% compared to nontreated, CMV-infected plants (Figure 2B). For PVX, the percentage of systemic infected leaves showed no statistically significant difference between treatments, except for strain R16, which gave lower percentages of infected leaves both at T1 (non-significantly) and T2 (significantly) (Figure 2C). In the case of PVY, all the plants exhibited similar numbers of systemic symptomatic leaves, and none of the treatments showed significant results, regardless of the treatment given to the infected plants (Figure 2D).

The severity of the symptoms was evaluated to describe the ability of the inoculated virus to cause symptoms when the plants underwent different treatments. The results obtained from this parameter were compared through a statistical analysis using a nonparametric Wilcoxon rank-sum test followed by *p*-adjusted values based on Bonferroni methods using the target viruses as the reference of comparison.

**Figure 3:**
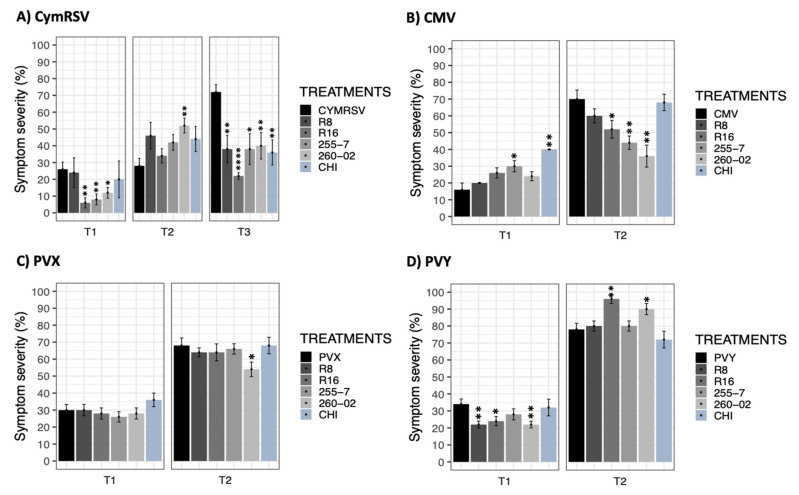
Bar graphs reporting the results based on mean percentage values of symptom severity. (**A**) CymRSV, (**B**) CMV, (**C**) PVX, and (**D**) PVY display the measurements taken in days post-inoculation (dpi) intervals represented as T1 = 5 dpi, T2 = 7 dpi, and T3 = 12 dpi. Each bar represents a different treatment: nontreated, virus-infected plants (CymRSV, CMV, PVX, and PVY) and virus-infected plants treated with either strain R8 [*Paraburkholderia*], R16 [*Paenibacillus*], 255-7 [*Pantoea*], 260-02 [*Pseudomonas*], or a chitosan-based product (CHI). Error bars indicate standard error. Asterisks indicate significant differences among the results according to a nonparametric Wilcoxon rank-sum test followed by Bonferroni methods compared with virus-infected, nontreated plants (* for *p* < 0.05, ** for *p* < 0.01, and **** for *p* = 0.000).

The plants infected with CymRSV showed varied results in the severity of the symptoms starting from T1, with statistically significant differences in the treatments with R16, 255-7, and 260-02 (I%I: 6–12%) compared to other treatments with no significant difference (I%I: 20–26%) (Figure 3A). Then, at T2, the severity of the symptoms in nontreated plants changed only slightly from the previous measurement, while the rest of the treatments showed extraordinary increment, especially in 260-02-treated plants (from 12% to 52%). However, an interesting result was obtained at T3, where the symptoms of CymRSV were fully developed on nontreated plants with a symptom severity of 72%, while all the other treatments managed to exert a statistically significant reduction in symptom severity, displaying as low as 22% in the case of strain R16 (Figure 3A). In the case of CMV-infected plants, the evaluation of symptoms at T1 showed a lower severity in nontreated plants and higher severity percentages in plants treated with the 255-7 strain and CHI (Figure 3B). At the second timepoint (T2), the symptoms of the nontreated plants were recorded as highest (70%) compared to the plants treated with strains R16, 255-7, and 260-02, which showed statistically significantly lower symptom severity for up to 36% (Figure 3B). The assessment of PVX-infected plants indicated nonsignificant symptom severity among all the treatments, regardless of ePGPB and chitosan inoculation, at all the timepoints apart from strain 260-02, which was able to significantly reduce the severity of the symptoms up to 54% at T2, while the nontreated plants showed percentages of symptom severity up to 68% (Figure 3C). The results obtained from PVY-infected plants at T1 revealed an incredible effect on the plants treated with strains R8, R16, and 260-2, showing 22–24% severity compared to other treatments, i.e., 28–34% (Figure 3D). However, at T2, plants treated with strains R16 and 260-2 gave significantly severe symptoms, even more than nontreated, PVY-infected plants, as shown in Figure 3D.

**Figure 4:**
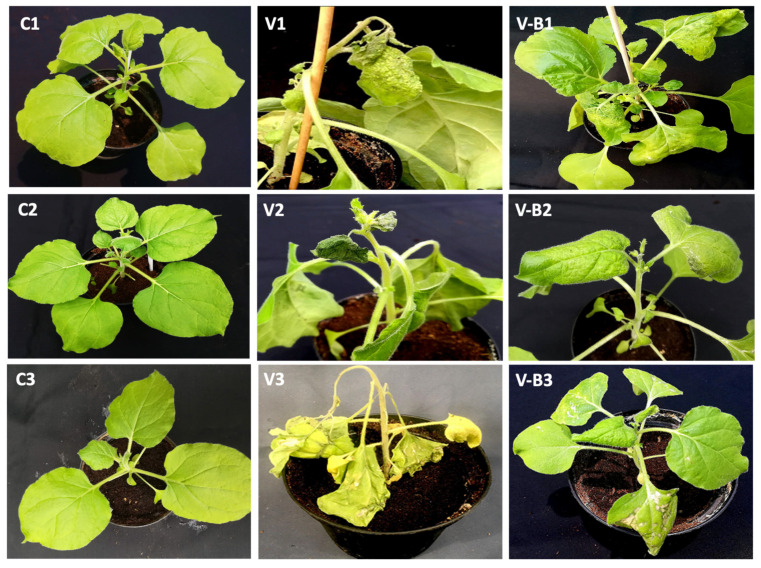

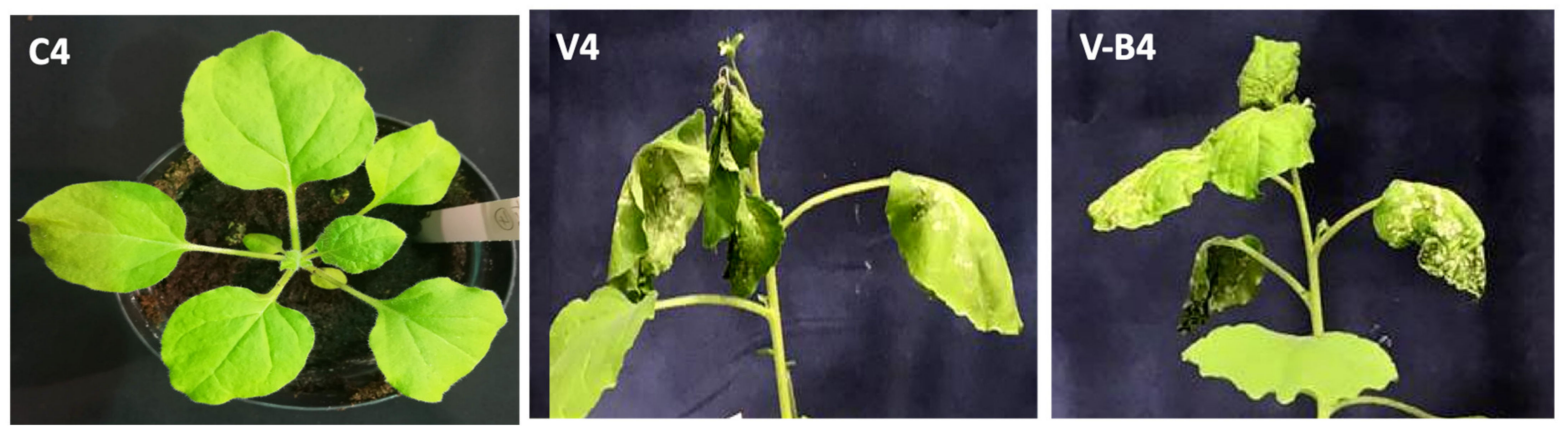
Photographs displaying the symptoms on *N*. *benthamiana* plants infected with CymRSV at T3 = 12 dpi and with CMV, PVX, and PVY at T2 = 7 dpi. (**C1**–**C4**) Nontreated control plants with no display of symptoms. (**V1**) Nontreated plant infected with CymRSV displaying severe symptoms of class 4, i.e., 72%. (**V-B1**) Plant treated with strain 255-7 showing milder symptoms of CymRSV infection of class 2, i.e., 38%. (**V2**) Nontreated plant infected with CMV displaying severe symptoms of class 4, i.e., 70%. (**V-B2**) Plant treated with strain R16 showing milder symptoms of CMV infection of class 3, i.e., 52%. (**V3**) Nontreated plant infected with PVX displaying severe symptoms of class 4, i.e., 68%. (**V-B3**) Plant treated with strain 260-02 showing milder symptoms of PVX infection of class 3, i.e., 54%. (**V4**) Nontreated plant infected with PVY displaying severe symptoms of class 4, i.e., 78%. (**V-B4**) Plant treated with strain R8 showing insignificant milder symptoms of PVY infection of class 4, i.e., 79.5%.

In accordance with these results, nontreated, CymRSV-infected plants displayed severe symptoms, causing crinkling, yellowing, and necrotic spots on inoculated leaves (Figure 4V1), whereas plants inoculated with strain 255-7 and infected with CymRSV displayed significant improvement and milder symptoms (Figure 4VB1). The symptoms of CMV infection were very intense and caused strong leaf crinkling, yellowing, mosaic patterns, and death of the systemic leaves (Figure 4V2). In contrast, the plants treated with the R16 strain and infected with CMV displayed slight improvement and milder symptoms after the infection (Figure 4VB2). As for PVX, the symptoms of both treated and nontreated plants were intense, and the infected plants exhibited necrosis, irregular spotting all over the leaves, stunted growth, and ultimately, died within 10 days post-inoculation (Figure 4V3). The only exceptions were the plants that were treated with strain 260-02, which presented significantly milder symptoms, as shown in the Figure 4VB3. The symptoms of PVY infection were severe, even in the plants treated with the ePGPB strains, causing overall irregular mosaic patterns, leaf crinkling, stunted growth, and death of the growing points of the plant (Figure 4V4). Even with the nonsignificant differences among treated and nontreated PVY-infected plants, the ePGPB strains showed minor improvements, as shown in Figure 4VB4 for strain 255-7, in comparison with the nontreated, infected plants.

#### 2.1.3. Relative Quantification of Virus

The relative quantification of the virus was carried out to discriminate whether the treatments influenced the ability of the virus to replicate in the host, reducing its concentration, or if they instead affected not its replication, but the pathogenesis mechanisms and plant defense pathways that could determine whether the symptoms developed or not.

**Figure 5:**
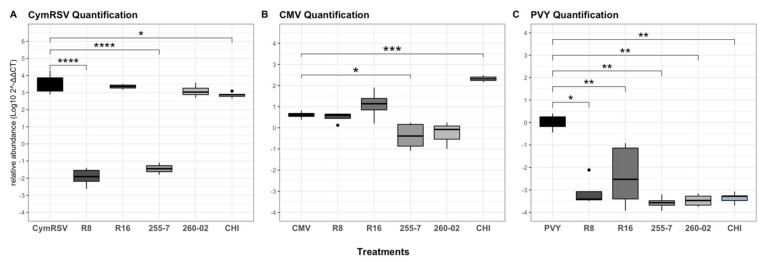
Boxplots indicating fold change (Log102^−ΔΔCT^) values of (**A**) CymRSV quantification under different treatments sampled at T3. Both (**B**) CMV quantification and (**C**) PVY quantification under different treatments sampled at T2. Asterisks indicate significant differences among the results according to the *t*-tests for CymRSV and CMV followed by *p*-adjusted values using Benjamini and Hochberg (BH) methods, as well as according to a nonparametric Wilcoxon rank-sum test for PVY followed by Bonferroni methods, compared with virus-infected, nontreated plants (* for *p* < 0.05, ** for *p* <0.01, *** for *p* <0.001, and **** for *p* = 0.000).

The results of the *t*-test gathered from the fold change (Log_10_2^−ΔΔCT^) values of the relative quantification of CymRSV at T3 demonstrated equal amounts of virus load present among all the treatments, except for the treatments of R8, 255-7, and CHI, which significantly lowered the concentration of the virus within the plants (Figure 5A).

While quantifying CMV, the results obtained from the *t*-test indicated that the concentration of the virus was significantly lower only in the leaves of plants treated with strain 255-7 compared to nontreated plants. It is interesting to note that, in the case of CHI-treated plants, the quantification of the virus was significantly higher than the treated plants, while in all other cases, the difference in concentration was always given by a decrease in concentration. All the other treatments had no statistically significant differences in virus concentration from nontreated plants (Figure 5B).

The results of the quantification of PVY as analyzed with a Wilcoxon rank-sum test were seen with great significant reduction in all the plants treated with both ePGPB strains and the chitosan-based product compared to nontreated plants (Figure 5C).

### 2.2. Effect of Symptom Severity on Virus Quantification

In order to understand if there was a correlation between symptom severity and the concentration of the virus in the plant, a linear regression analysis was performed using the percentage of symptom severity as the independent variable and Log_10_2^−ΔΔCT^ values of virus quantification as the dependent variable at T3 for CymRSV and at T2 for CMV and PVY.

**Figure 6:**
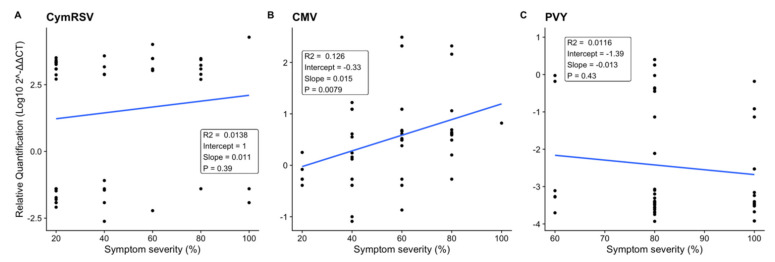
Regression analysis representing the effect of the percentage of symptom severity on the *x*–axis and the Log_10_ ratio of virus quantification on the *y*–axis in each treatment: (**A**) CymRSV-infected plants at T3 = 12 dpi and (**B**) CMV-infected plants and (**C**) PVY-infected plants at T2 = 7 dpi. R-squared values present the goodness-of-fit measure of the model, and *p*-values show the significant difference (*p* < 0.05) between each treatment.

The results obtained for CymRSV (*p* = 0.39, R^2^ = 0.0138) and PVY (*p* = 0.43, R^2^ = 0.0116) indicated that there was no statistically significant correlation between the symptoms observed on a plant and the quantity of the virus detected, since samples with the same symptom severities could have either a very high or very low abundance of the virus (Figure 6A,C). This was not the case for CMV samples, which showed a statistically significant (*p* = 0.008), if weak (R^2^ = 0.126), correlation between increases in the concentration and the severity of symptoms (Figure 6B).

### 2.3. Effect of ePGPB Strains on Defense-Related Genes

The evaluation of gene expression was conducted using three treatments: (i) NT-C, (ii) CHI-C, and (iii) plants treated with ePGPB strains (R8, R16, 255-7, and 260-02). The leaf samples were harvested at 24 h post-inoculation prior to inoculation with the respective virus. This analysis allowed the evaluation of the regulation of plant-defense-related genes by the treatments in the absence of a pathogen. Statistical comparisons were made between the different treatments in reference to NT-C using transformed Log_10_2^−ΔΔCT^ values based on three defense genes (*NPR1*, *PR2B*, and *EDS1*). Significant differences were determined using a nonparametric Wilcoxon rank-sum test followed by a Bonferroni test after checking the normal distribution of the data and the homogeneity of variances among the treatments.

**Figure 7:**
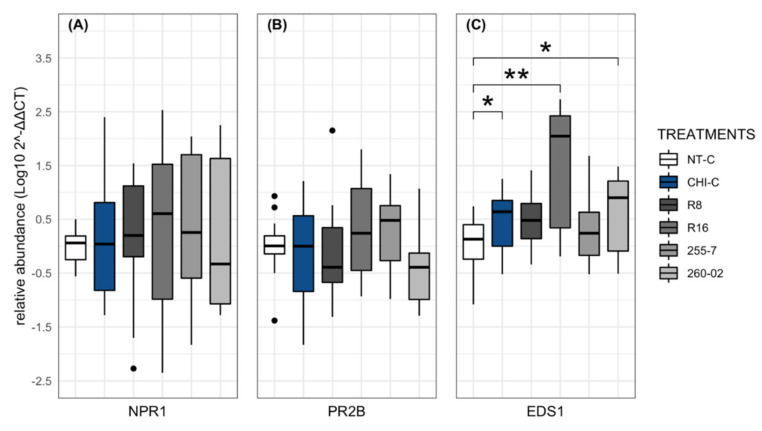
Relative gene expression assay in *N*. *benthamiana* plants (treated and nontreated) with ePGPB strains and chitosan indicator (CHI-C) sampled at 24 hpi: (**A**) *NPR1*, (**B**) *PR2B*, and (**C**) *EDS1*. The y–axis represents 2^−ΔΔCT^ value expressed as the Log_10_ of each gene normalized on the PP2A gene, and x–axis represents different treatments. Bars represent the standard error of mean values at a 95% confidence interval. Asterisks indicate significant difference according to a nonparametric Wilcoxon rank-sum test followed by *p*-values based on Bonferroni methods compared with nontreated group (* for *p* < 0.05 and ** for *p* < 0.01).

The results obtained from the *NPR1* and *PR2B* genes indicated no significant differences among the treatments compared to the nontreated control (Figure 7A,B). Interestingly, the *EDS1* gene underwent upregulation in the plants treated with chitosan, as well as with strains R16 and 260-02, when compared to the nontreated control (Figure 7C).

## 3. Discussion

Endophytic plant-growth-promoting bacteria (ePGPB) have been utilized for years as a means to promote plant growth and reduce the damage caused by bacterial and fungal pathogens with a lower environmental impact than synthetic products, but their potential against viral diseases has not been explored much until recently [56]. In this perspective, we investigated four ePGPB strains (R8, R16, 255-7, and 260-02), some of which have already been characterized for their potential to enhance plant growth and ameliorate responses to various biotic stresses [44,45,46], against four phytopathogenic viruses (CymRSV, CMV, PVX, and PVY) on *N*. *benthamiana* plants. In addition, the treatments were compared with a chitosan-based product (CHI-S), as these molecules are known to induce resistance in plants. Our objective was to evaluate the defense-priming actions of the investigated ePGPB strains, their plant growth promotion capabilities, and a possible mechanism of action behind the biocontrol effect that was registered towards the respective virus.

While considering the phenotypic profile, all four ePGPB strains showed positive influences on growth promotion that not only allowed CymRSV-infected plants to grow to heights comparable to noninfected controls, but also showed a growth promotion potential to reduce the effects of CMV and PVX (except for strain R8). These findings are in agreement with Kumar et al. [38], who reported the growth promotion of CMV-infected tobacco plants with the application of the *Paenibacillus lentimorbus* B-30488 strain in the soil. It was more evident in the case of strain 260-02, as the present study agrees with previous findings that demonstrated its ability to promote growth in bell pepper and tomato plants, other plants of the Solanaceae family, and gave preliminary data regarding its potential antiviral activity towards CymRSV [46]. It is notably promising that these strains exert their growth-promoting capabilities even under various biotic stresses. Still, the plant growth promotion of the strains was less in plants infected with CMV and PVX, suggesting that the symptoms caused by these viruses may interfere with the strains’ abilities to promote plant growth. A similar phenomenon was registered on the plants infected with PVY, where none of the strains contributed to plant growth promotion.

On the other hand, the effectivity of chitosan treatments to promote growth was confined to CMV and PVX, and they were not effective in CymRSV-infected and PVY-infected plants. The results of chitosan against CMV and PVX are in accordance with previous studies [23,57] that have reported improvement in the vegetative growth of tomato and cucumber plants infected with Tomato Mosaic Virus and Squash Mosaic Virus, respectively.

The parameters evaluated to describe the progress and severity of viral infection in the host plants were: (i) the percentage of leaves systemically infected, indicating the spread of the virus inside the host plant; (ii) the symptom severity, indicating the ability of the virus to produce symptoms in the host plant; and (iii) the virus concentration, indicating the ability of the virus to reproduce in the host plant. The first two phenotypic parameters were in accordance among all the treatments and viruses. While the values of the two indexes may not be similar or follow a specific trend, a statistically significant reduction in comparison to the nontreated control in one parameter was always also accompanied by a statistically significant reduction in the other. This result suggests that the overall amelioration of the host plant health contributed both to counteract the expression of symptoms and the diffusion of the virus inside the host.

The results obtained regarding strain 260-02 are in accordance with and add to those described by Passera et al. [46] in which the biocontrol effect of strain 260-02 in pepper plants mechanically inoculated with CymRSV was reported. The present study extends these results to a new host (*N*. *benthamiana*) and new pathogens (CMV and PVX).

The treatments with the commercial chitosan-based product (CHI) proved to be effective in reducing the symptoms of CymRSV but not against CMV-, PVX-, and PVY-infected plants. The results were found to be both in accordance (in the case of CymRSV) and in contradiction (in the cases of CMV, PVX, and PVY) with studies conducted by Chirkov and colleagues [57], as they reported that spraying with chitosan provided resistance in potato plants against viral infection. Nevertheless, the results of CMV and PVX are in accordance with those presented by Kumar and his co-workers [38], which reported that chitosan resistance inductors led to a significantly higher plant height in CMV-infected plants but not to a significant reduction in symptoms. To the best of our knowledge, the effect of chitosan treatments against CymRSV is reported for the first time in this study.

In this study, all the viral pathogens started to manifest symptoms at 5 days after inoculation in all the plants, both treated and nontreated. Several previous works, carried out both using bacterial biocontrol agents and chitosan-based products, have reported that part of the biocontrol effect was a delay in symptom manifestation that could range from 11 to 20 days [31,38,58]. The difference between the results of this study and the above-mentioned results could be due to the use of different bacterial strains, chitosan formulations, viral strains, or even different plant species. Additionally, *N*. *benthamiana* has been utilized as a model host plant for viral infection especially for its impressive susceptibility against a variety of plant viruses compared to that of natural host plants [59,60,61].

The third parameter, virus quantification, instead did not show a clear correlation to the phenotypic parameters and could give rise to different cases: (i) a reduction in symptom severity accompanied by a decrease in virus concentration (for example, CymRSV-infected plants treated with R8, 255-7, or CHI and CMV-infected plants treated with strain 255-7); (ii) a reduction in virus concentration not accompanied by a decrease in symptom severity (for example, PVY-infected plants treated with strains R16 and 260-02); or (iii) the opposite, in which the concentration of the virus was not reduced, but the symptom severity was greatly reduced (for example, CymRSV- and CMV-infected plants treated with strains R16 and 260-02). The most unexpected results recorded were that (iv) there were no differences in symptoms compared to the nontreated control, but the concentration of the virus was much higher (CMV-infected plants treated with CHI) or (v) the concentration of the virus was much lower (PVY-infected plants treated with bacterial strains).

The effect and relevance of virus concentration on the symptoms caused is still a matter of debate in the scientific community, with published studies showing both that there is a correlation between the two parameters [62] and that no correlation is present [31,63]. The present study reinforces the idea that no strict correlation is present between virus concentration and symptom severity, but also, this result could be dependent on the particular combination of biocontrol agents, viral strains, and plant host utilized. Considering these results about viral concentration and symptom severity, which do not show correlation, discrepancies in the effects of the endophytes on symptom severity and viral replication are plausible: both in cases where the reduction in symptoms was accompanied by a reduction in viral concentration (such as CymRSV-infected plants treated with R8) and where the reduction in symptoms was not accompanied by this phenomenon (such as CymRSV-infected plants treated with R16) it can be concluded that interference with viral replication was not a mechanism of action directly underlying the biocontrol effect.

The last objective of the present study was to understand a possible mechanism of action behind the biocontrol effect that was registered toward the viruses. Considering the unique nature of viruses, the most likely biocontrol trait involved was the induction of the host plant’s defenses. The elicitation of defense-related genes mediated by ePGPB has been studied in numerous studies to understand which signaling pathways are associated with the induction of systemic plant resistance [64,65]. The present study analyzed a set of three defense-related genes [66]: *EDS1*, an upstream gene in the salicylic acid (SA) signaling pathway [67]; *NPR1*, a master regulator gene that mediates the crosstalk between the pathways related to SA or jasmonic acid (JA) and ethylene (ET) [68]; and *PR2B*, a pathogenesis-related protein that is a molecular marker of systemic acquired resistance (SAR) [69].

The results obtained from the plants treated with strains R16 and 260-02, which gave the greatest reduction in symptom severity for CymRSV and CMV, respectively, showed an upregulation of the *EDS1* gene without having a significant impact on the *NPR1* and *PR2B* genes compared to nontreated control plants. These results suggest that the mechanism involved could be related to SA-dependent ISR pathways, as reported previously for other strains belonging to the genera *Paenibacillus* and *Pseudomonas* [41,70]. Furthermore, Beris and colleagues [39] demonstrated that the activation of SA-independent defense pathways by a cell-free culture filtrate of *Bacillus amyloliquefaciens* strain MBI600 did not trigger *NPR1* and *EDS1* gene expression activation in tomato plants. This could be an explanation for strains R8 and 255-7, which did not show upregulation for any of the investigated genes. This might also suggest that they either affected different pathways in the plant host or only acted as plant-growth-promoting agents, providing a benefit to plant health that was dependent on nutrition only. However, it must be considered that the application of ePGPB in uninfected plants does not necessarily induce defense-related gene expression; instead they often triggered with the pathogens and pests attack [41]. Therefore, carrying out gene expression analyses on plants that receive both bacterial and virus inoculum could better unveil the hallmarks of ISR facilitated by ePGPB but, at the same time, add a layer to the interaction, making the results more difficult to analyze and interpret.

Regarding the chitosan-based resistance inducer, CHI-C showed an upregulation in the *EDS1* gene only, apparently having the same effect as the treatments with strains R16 and 260-02, suggesting the activation of a SA-dependent pathway. These results are in contradiction with those reported by Chirkov [71], as well as Redina and colleagues [58], but are in accordance with the findings of Beatrice and colleagues [72], which indicated the higher expression levels of PR1 and PR5 genes through chitosan treatment in kiwifruit plants and, hence, SA-mediated pathway involvement.

## 4. Materials and Methods

### 4.1. Plant Material and Microbial Strains

#### 4.1.1. Plant Material

Plants of *Nicotiana benthamiana* were used as a model host for the inoculation of different pathogenic and biocontrol agents. The seeds were sown 3 weeks before the experimental trials under greenhouse conditions (25 °C, 72% RH, 16/08 h light/dark photoperiod) at the Department of Agricultural and Environmental Sciences—Production, Landscape, and Agroenergy, University of Milan, Milan, Italy.

#### 4.1.2. ePGPB Strains

Four endophytic plant-growth-promoting bacteria (ePGPB) strains (*Paraburkholderia fungorum* strain R8 [73], *Paenibacillus pasadenensis* strain R16 [44,73], *Pantoea agglomerans* strain 255-7 [74], and *Pseudomonas syringae* strain 260-02 [45]) were utilized. All the strains were cultured in a lysogeny broth (LB) high-salt agar (LBA) plated medium (10 g/L tryptone, 5 g/L yeast extract, 10 g/L sodium chloride, and 15 g/L agar) at 24 °C for a short-term period and maintained in an LB: glycerol (7:3) solution at -80 °C for long-term conservation.

#### 4.1.3. Chitosan-Based Resistance Inductor

A chitosan-based product, “ChitoPlant Solution” (CHI-S), obtained from Agritalia, Mantua, Italy, was used as a positive reference of resistance induction. Following the manufacturer instructions, a homogenous chitosan solution was prepared by adding 3 mL of the CHI-S product into 147 mL of distilled water, with a final concentration of 1:50 *v*/*v*.

#### 4.1.4. Phytopathogenic Viral Strains

Four plant pathogenic viral strains (Cymbidium Ring Spot Virus PV-0272 (CymRSV), Cucumber Mosaic Virus PV-0504 (CMV), Potato Virus X PV-0017 (PVX), and Potato Virus Y PV-1036 (PVY)) were bought from the Deutsche Sammlung von Mikroorganismen und Sellkulturen GmbH (DSMZ, Germany).

The plant pathogenic viral strains were propagated onto 3-week-old *N*. *benthamiana* seedlings (10 biological replicates per virus) following the DSMZ virus inoculation protocol with some modifications. Briefly, freeze-dried leaves containing the viruses were ground in a mortar with 0.05 M sodium/potassium phosphate (Norit) buffer (pH 7, containing DIECA at 5 mM and EDTA at 1 mM) at a final concentration of 1:10 *w*/*v* and inoculated mechanically onto the tops of 3 fully developed leaves of *N*. *benthamiana* seedlings after the application of abrasive carborundum spray. The following symptoms were observed at two weeks post-inoculation: for CymRSV, necrotic spots, yellowing, crinkling of leaves, and stunted growth; for CMV, irregular mosaic patterns, necrotic areas, crinkling of leaves, and stunted growth; for PVX, interveinal chlorosis, leaf crinkling, mosaic patterns, necrosis, and stunted growth; and for PVY, mosaic patterns or mottling, crinkling, and stunted growth. Samples of leaves exhibiting systemic infection were harvested, quickly frozen with liquid nitrogen, and stored at −80 °C to be used as inoculum source for the viruses in the following assays.

### 4.2. Plant Growth Promotion, Biocontrol, and Defense-Related Gene Expression Bioassays

#### 4.2.1. Experimental Setup

A total of 5 treatment groups containing different biological replicates were employed in two sets of bioassays: (1) a plant growth promotion and biocontrol assay and (2) a plant-defense-related gene expression assay.

The groups were comprised of (i) nontreated control plants (NT-C); (ii) plants infected mechanically with viral inoculum only (CymRSV, CMV, PVX, or PVY); (iii) plants treated with ePGPB inoculum and then infected with viral inoculum (R8, R16, 255-7, or 260-02); (iv) plants treated with CHI-S inductor only (CHI-C); and (v) pretreated CHI-S plants infected with respective viral inoculum (CHI). The numbers of plants as biological replicates utilized by each treatment with regard to each bioassay are listed in Table 1. This setup was employed for each virus assessed in the study.

#### 4.2.2. Inoculation of Plants with ePGPB Strains and Chitosan-Based Product

A liquid suspension of ePGPB inoculum was prepared as described in Passera et al. [46]. Briefly, for each strain, 3 mL of LB was inoculated with a single, actively growing colony and incubated overnight at 24 °C on an orbital shaker at 230 rpm. The next morning, 1 mL of the culture media was transferred to a sterile 500 mL conical flask containing 100 mL of LB broth and left for an 8 h incubation period under the same conditions. The bacterial cells were then pelleted through centrifugation at 4000 rpm for 10 min, resuspended in sterile Ringer’s solution (Sigma-Aldrich, St. Louis, MO, USA), and diluted to the desired concentration of 10^6^ CFU/mL.

The bacteria were inoculated with soil drenching, applying 20 mL of the respective liquid suspension of ePGPB inoculum to 7 cm diameter pots containing three-week-old *N*. *benthamiana* seedlings grown in potting soil. The same amount of sterile Ringer’s solution was soil-drenched for the rest of the non-ePGPB-treated plants.

At 6 days post-ePGPB-inoculation, an aqueous solution (CHI-C) of chitosan-based product (CHI-S) was freshly prepared following the manufacturer instructions, as mentioned in Section 2.1.3. An amount of 1 mL of CHI-C was dispensed using foliar spraying onto the non-ePGPB-treated *N*. *benthamiana* seedlings (1 plant per pot).

One day after the drenched inoculation of the ePGPB strains or the foliar-sprayed treatment with CHI-C, leaf samples (0.5 g) were collected from five of the inoculated plants for each strain and the nontreated control (NT-C) to be used for RNA extraction and the quantification of gene expression (Section 4.3.2).

#### 4.2.3. Virus Inoculation, Phenotypic Analysis, and Leaf Sampling

The viral strains were mechanically inoculated, as previously described, on the *N*. *benthamiana* plants (10 biological replicates of treated and nontreated plants for each ePGPB strain and 5 replicates of treated and non-treated plants for chitosan inductor) on the day (T0) that corresponds to one week after bacterial inoculation [58,75] and one day after treatment with chitosan [58]. After inoculation, the plants were monitored for the following parameters: plant height, number of systemically infected leaves, and symptom severity. The plant height was measured from the surface of the soil to the top node in the plant’s stem, not considering any foliar laminae or flower buds that reached greater heights. The rate of systemically infected leaves was measured as the percentage of symptomatic leaves developed above the virus inoculation site to the total number of leaves present above the virus inoculation site. The symptom severity was measured by attributing each plant to a symptom severity class from 0 to 5 (where 0 = no symptom; 1 = mild leaf curling; 2 = moderate leaf curling, mild yellowing, and necrotic spots or mosaic pattern; 3 = strong leaf curling, moderate yellowing, necrotic spots or mosaic pattern, and mild stunted growth; 4 = strong leaf curling, yellowing, necrotic spots or mosaic pattern, and stunted growth; 5 = death of the plant) (Figure 4). The symptom classes were later converted to an infection percentage index (I%I) using the formula presented by Townsend and Heuberger [76]. The plants infected with CymRSV were inspected at three different times: T1, 5 days post-infection (dpi); T2, 7 dpi; and T3, 12 dpi. The plants infected with CMV, PVX, and PVY were inspected only at T1 and T2. CymRSV was observed for a longer time because it needed more time to develop symptoms of intensity comparable to those of the other three pathogens (Figure 3). The plant heights were also recorded at the day of inoculation (T0).

### 4.3. Relative Quantification of RNA

From all the collected samples, both for plants sampled one day after ePGPB or chitosan treatments (Section 4.2.2) and for those infected with the viruses (Section 4.2.3), the total RNA was extracted using the 2% CTAB method described by Gambino et al. [77]. The extracted RNA was employed for the relative quantification of selected, relevant transcripts. Regardless of the target, the same workflow was used: starting from 1 µg of RNA, real-time PCR assays were performed using a two-step SYBR^®^ Green approach in a StepOnePlus™ thermocycler (Thermo Fisher Scientific, Waltham, MA, USA). The cDNA synthesis was carried out using a GoScript™ Reverse Transcription System (Promega Corporation, Madison, WI, USA) kit. Following the manufacturer instructions, reverse transcription PCR was performed in a 20 μL total volume reaction using 5 μL of 0.2 μg/μL random examer primer, 1 μL of 10 mM dNTPs, 2 μL of 0.1 M dithiothreitol (DTT), 2 μL of 10× Retro-transcription Buffer, 2 μL of 50 μM MgCl_2_, 1 μL M-MuLV (Moloney murine leukemia virus) reverse transcriptase enzyme, and water to reach the required volume. The reaction was carried out with the following thermal cycle: 25 °C for 10 min, 37 °C for 60 min, 70 °C for 5 min, and then kept at 4 °C. For qRT-PCR, each reaction was carried out at a 20 μL volume containing 2 μL of aliquot of respective virus cDNA, 0.4 μL of 400 μM reverse/forward primer, 5 μL of 1x Power SYBR^®^ Green PCR Master Mix (Thermo Fisher Scientific, Waltham, MA, USA), and water to reach the total volume of 20 μL. Each sample was amplified in triplicate to obtain a more precise value for the threshold cycle.

The obtained Ct values from each treatment were normalized with endogenous plant gene PP2A using the 2^−ΔΔCT^ method described by Livak and Schmittgenin [78]. The Log10 transformation was performed at 2^−ΔΔCT^ for the optimization of the data scale.

#### 4.3.1. Relative Quantification of Virus

The viral strains utilized in this study were quantified using specific primer pairs. The details of the corresponding primer sequences of each viral strain, along with their targeted gene and fragment lengths, are reported in Table 2.

For the 2^−ΔΔCT^ method, a nontreated, virus-infected sample was selected as the reference for the calibration of the data.

#### 4.3.2. Relative Quantification of Defense-Related Genes

Real-time PCR assays were carried out to evaluate the expression levels of three selected defense genes (*Enhanced Disease Susceptibility-1*, referred to as *EDS1*; *Non-expressor of Pathogenesis-related genes-1*, referred to as *NPR1*; and *Pathogenesis-related protein-2B*, referred to as *PR2B*). All the primers utilized in this assay are reported in Table 3. For the 2^−ΔΔCT^ method, a nontreated sample was selected as the reference for the calibration of the data.

### 4.4. Statistical Data Analysis

All the data collected from the above-mentioned bioassays were subjected to statistical analysis using R-studio, version 3.6.1 (2019-07-05). In particular, the data obtained for the plant heights (described in Section 4.2.3) were analyzed with a Kruskal–Wallis ANOVA test, followed by *p*-adjusted values using Benjamini and Hochberg (BH) methods. The data obtained from the percentage of symptomatic leaves and the percentage of symptom severity (described in Section 4.2.3) were analyzed with a nonparametric Wilcoxon rank-sum test, followed by *p*-values using Bonferroni methods. The relative quantification data of the viruses and gene expressions of CymRSV and CMV (Section 4.3.1) were analyzed with parametric *t*-test with *p*-adjusted values using Benjamini and Hochberg (BH) methods for data that were normal and homogeneous (CymRSV and CMV quantification; Section 4.3.1). A nonparametric Wilcoxon rank-sum test with *p*-values using Bonferroni methods was used for those datasets that did not show normality or homogeneity (PVY quantification and defense-related genes; Section 4.3.1 and Section 4.3.2). For all the above-mentioned data analyses, the degree of normality of the data was checked using Shapiro–Wilk’s normality test, followed by Levene’s test for the homogeneity of variances among the treatments to choose between parametric or nonparametric tests. Furthermore, to determine the correlation effect of symptom severity on virus quantification, a simple linear regression analysis was performed using a linear model function.

## 5. Conclusions

This study provided data on the efficacy of four different ePGPB strains against four different viruses on the model host plant *N*. *benthamiana*. The range of the obtained results, including a great reduction in symptom severity against CymRSV and CMV but small results against PVX and PVY, showed that there is promise for the use of this biocontrol technique against viral diseases, but there is also much more to learn. While the study of relative gene expression revealed correspondence in the impact that ePGPB strains had on the host plant, there are still many variables that need to be considered and dissected. Future studies are needed to clear several questions that remain open regarding this biocontrol effect, such as (i) employing the same study on the natural host plants of the respective viruses to verify whether the effectiveness of the investigated ePGPB treatments is greater in species that are less sensitive to viruses than model host plant; (ii) investigating how long the protection is afforded by treatment with ePGPB strains, (iii) investigating in planta real-time imaging to visualize the movement and endophytic colonization patterns of the investigated strains that undergo biocontrol activity against the respective viruses, (iv) evaluating the epidemiological implications of these treatments, considering that the plants showing milder symptoms can still act as sources of inoculum in the field, and (v) going more in-depth in the study of the gene expression patterns associated with the treatments by analyzing more genes (RNA-seq), considering more timepoints, and including the plant–ePGPB–virus interactions in the experiment to describe the plant’s response and fill the major gaps. While these experiments can help to determine more precisely the nature, extent, and mechanisms of biocontrol, the present study already proved the effectiveness of four new bacterial strains in protecting plants from viral pathogens.

## Figures and Tables

**Table 1:** List of all the biological replicates within the different treatment groups for biocontrol and defense-related gene expression assays.

Treatments	Biocontrol Assay (n#)	Defense-Related Gene Expression Assay (n#)
NT-C	10	5
CymRSV, CMV, PVX, PVY	10	-
R8, R16, 255-7, 260-02	10	5
CHI-C	5	5
CHI	5	-

**Table 2:** List of the primers used for the quantification of viruses performed with qPCR assays.

Primer Pair	Sequence 5′-3′	Fragment (Target Gene)	Bibliography
PP2A—forward	GACCCTGATGTTGATGTTCGCT	123bp	[59]
PP2A—reverse	GAGGGATTTGAAGAGAGATTTC		
CymRSV—forward	GTA CAT GCG TCA CTT GGG GA	195bp (RNA polymerase)	[79]
CymRSV—reverse	TCT CAG CAT CTT CCA ACC GC		
CMV—forward	CTG GCG ACA ATC TGG AGG GA	153bp (Movement protein)	[80]
CMV—reverse	CGA TAA CGA CAG CAA AAC AC		
PVX—forward	GCC CAA TTG TTA CAC ACC	101bp (Coat protein)	[81]
PVX—reverse	CTA GCC TCA TCT TAA TG		
PVY—forward	GGT AGC ACA ACT ATA CGG TGC	100bp (Coat protein)	[82]
PVY—reverse	GAT GTT TGG GGT CGA TCC A		

The first column reports the name of the primer; the second, the sequence of each primer; the third, the fragment length with the targeting protein; and the fourth, the reference from which the primer was obtained.

**Table 3:** List of all the primers used for the relative quantification of gene expression analysis performed with qPCR assays.

Primer Pair	Sequence 5′-3′	Fragment	Bibliography
PP2A—forward	GACCCTGATGTTGATGTTCGCT	123 bp	[59]
PP2A—reverse	GAGGGATTTGAAGAGAGATTTC		
*NPR1*—forward	GGC CTT GCC TCA TGA TAT TG	187 bp	[66]
*NPR1*—reverse	GCT ACA GCA TAA TGG AGA GC		
*PR2B*—forward	CTAAAGAGGGTAGCCCAAGA	147 bp	[66]
*PR2B*—reverse	GTCCCAAACTCCACCAGAGA		
*EDS1*—forward	GGACAATGGGAGAAGCAGAA	118 bp	[66]
*EDS1*—reverse	GAACGCATCATAATACCCGA		

The first column reports the name of the primer; the second, the sequence of each primer; the third, the fragment length with the targeting protein; and the fourth, the reference from which the primer was obtained.

## Data Availability

All data produced in this study can be found in the main text, figures, and tables of the manuscript.
